# Supplementary figures and images for: Phylogenetic and divergence analysis of Pentatomidae, with a comparison of the mitochondrial genomes of two related species (Hemiptera, Pentatomidae)
Source: PLoS One. 2024 Oct 23;19(10):e0309589. doi: 10.1371/journal.pone.0309589 (PMC11498689; doi:10.1371/journal.pone.0309589)

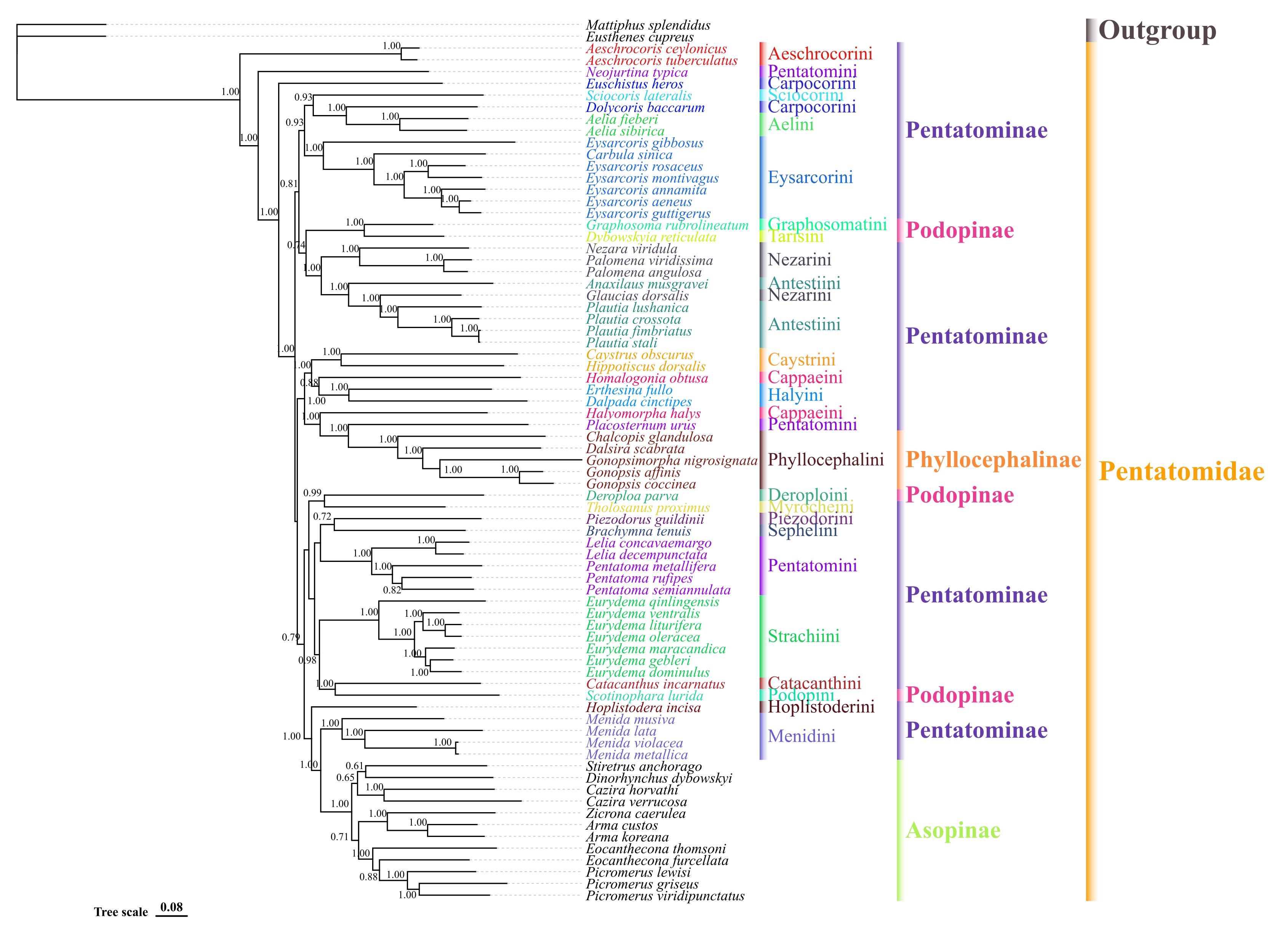

Supplement: S1 Fig — Numbers on nodes are the posterior probabilities (PP), lower than 0.6 is not displayed. (JPG) [file pone.0309589.s005.jpg]
