# Supplementary material for: Phylogenetic and divergence analysis of Pentatomidae, with a comparison of the mitochondrial genomes of two related species (Hemiptera, Pentatomidae)
Source: PLoS One. 2024 Oct 23;19(10):e0309589. doi: 10.1371/journal.pone.0309589 (PMC11498689; doi:10.1371/journal.pone.0309589)
Supplement: Attachment — Submitted filename: Response to Reviewers[PONE-D-24-13214].docx [file pone.0309589.s006.docx]

Dear Editor,

Thanks for your letter and for reviewers’ comments concerning our manuscript entitled “Phylogenetic and divergence analysis of Pentatomidae, with a comparison of the mitochondrial genomes of two related species (Hemiptera, Pentatomidae)”. These comments are all valuable and helpful for revising our manuscript. We have read through comments carefully and have made corrections. Revisions in the text are shown using red highlight for additions, and strikethrough font for deletions. The responses to the reviewer's comments are marked in red and presented following.

Thank you for reviewing again and we look forward to hearing from you.

Sincerely,

Qing Zhao

First of all, we made a statement on the following points.

1. We have ensured that the manuscript format conforms to the style of plos one.

2. The funders had no role in study design, data collection and analysis, decision to publish, or preparation of the manuscript.

3. The authors have declared that no competing interests exist.

4. We have revised and proofread the references on page 8 according to your suggestions.

**Response to Reviewer #1**

Point 1: The manuscript needs to more discussion on the estimated divergence time between genera/subfamilies.

**Response:** **Thank you for the reviewer's comments. We have added further discussion on the estimated divergence time between genera/subfamilies in the manuscript.**

Point 2: Figures and tables or supporting information should be cited adequately in the section of Discussion for better interpretation.

**Response:** **Thanks to the reviewer's suggestion, we added the citation in the corresponding position in the manuscript.**

Point 3 ： Please consider the all abbreviations across the manuscript.

**Response: Thanks to your suggestions. We double-checked all the abbreviations in the manuscript.**

Point 4： Some parts of the manuscript have been repeated and the same explanation is also included in the result/discussion section. It therefore needs to be shortened and discussed more comprehensively considering the relevant literature.

**Response: Thank you for the reviewer's comments.** **We have shortened and revised the discussion section.**

**Response to Reviewer #2**

Major comments:

Point 1: This manuscript needs extensive English editing. Some paragraphs and sentences need to be completely rewritten.

**Response: Thanks to your suggestion, we have rewritten some paragraphs and sentences in the manuscript.**

Point 2: The Introduction section should add more sentences to describe the phylogenetic conflicts or somewhat reflect your contributions in this work.

**Response:** **Thank you for your advice. In the Introduction, we have added Pentatomidae phylogenetic conflicts of previous researchers' findings, and described the content and contributions of the entire manuscript study in the last paragraph.**

Point 3: I suggest that the description of mitochondrial genome structure and characteristics be appropriately reduced in the Discussion section.

**Response:** **Thanks to your suggestions, I have appropriately reduced the description of mitochondrial genome structure and characteristics in the Discussion section.**

Minor comments:

Point 1: Line 25: "Pentatomidae, the most diverse subfamily of Pentatomoidea"; however, " Pentatomidae" is a family name, not subfamily.

**Response:** **Thank you for pointing out the problem, I have corrected it.**

Point 2: Line 27: Caution in the use of "controversial" in phylogenetic studies.

**Response: Thanks to your suggestions, I replaced “controversial” with “unstable” in the manuscript.**

Point 3: Line 31: "divergence time of Pentatomidae" instead of "Pentatomidae divergence time"; "Trees" instead of "trees"; "reconstructed" instead of "constructed".

**Response: Thanks to your suggestions, I have corrected it in the manuscript.**

Point 4: Line 32: "Divergence time of Pentatomidae"

**Response: Thank you, I have corrected it in the manuscript.**

Point 5: Line 40: Delete "(95% highest probability density: 138.38-83.77 Ma)", this information is not required to be included in the Abstract section.

**Response: Thanks to your suggestions, I deleted it in the Abstract section of the manuscript.**

Point 6: Lines 42-43: How do you think your results were more accurate?

**Response:** **We selected all currently applicable fossil information of Pentatomidae, including the calibration of stem group nodes and crown group nodes, to further study the divergence time within Pentatomidae more accurately.**

Point 7: Line 48: superfamily Pentatomoidea.

**Response:** **Thanks for your suggestion, I have corrected it in the manuscript.**

Point 8: Lines 50-53: You described the various body sizes of pentatomid members; however, I do not understand the connection between body size and mitogenomes or phylogeny.

**Response:** **Thank you for your question. In the manuscript, we describe the** **various body sizes of pentatomid members, focusing on the rich diversity of the species, followed by a phylogenetic analysis based on the use of mitochondrial genome data.**

Point 9: Line 62: "nuclear genes" instead of "molecular genes".

**Response: Thanks for your suggestion, I have corrected it in the manuscript.**

Point 10: Lines 60-76: Should be completely rewritten. You listed many previous works but just emphasize the monophyly of Pentatomidae in some studies, is it because its monophyly has not yet been determined? What is the connection between these works and your focused taxa in your study?

**Response:** **Thanks for your suggestion, I have corrected Lines 60-76 in the manuscript. This study elucidates the phylogenetic position of *Lelia*, and further research is needed on the monophyly of Pentatomidae.**

Point 11: Lines 67 and 75: Rephase the statement of "between the family Pentatomidae and Phyllocephalini" and "between Pentatominae and Menida". They are not the same level in taxonomy.

**Response: Thank you for your suggestion. I have rewritten this section.**

Point 12: Line 167: "PhyloSuite".

**Response: Thank you for pointing out the problem, I have corrected it.**

Point 13: Lines 297-300: Phylogenetic trees were reconstructed based on two datasets, and only one tree was selected as your main phylogenetic hypothesis. I noticed that the topologies of these two trees were distinct. Try to explain the reason why you selected the "PCG" topology.

**Response:	We consider the length of the whole manuscript and select the “PCG” topology as a representative to further construct the divergence time tree based on the PCG dataset.**

Point 14: Lines 310-311: Do you think the non-monophyly is due to the limited mitogenomic data?

**Response: I think the limited mitogenomic data will affect the judgment of monophyly. Animal mitochondrial genome has many common characteristics, such as stability of gene composition, relatively conservative gene arrangement, common maternal inheritance, and rarely recombination^[1]^, so it has been widely used in evolutionary and phylogenetic studies^[2]^. The limited mitochondrial genome data may not be fully representative of the genetic diversity of the study subjects. This may affect the judgment of monophyly in phylogenetic results.**

**Reference:**

**[1] Wolstenholme D R. Animal mitochondrial DNA: structure and evolution. International review of cytology, 1992, 141: 173-216.**

**[2] Wilson A C, Cann R L, Carr S M, et al. Mitochondrial DNA and two perspectives on evolutionary genetics. Biological Journal of the Linnean Society, 1985, 26(4): 375-400.**
